# Seroprevalence of Pediatric Malaria in Quetta, Balochistan, Pakistan

**Published:** 2013

**Authors:** K Hussain, M Shafee, N Khan, S Jan, AM Tareen, MA Khan

**Affiliations:** 1 Microbiology Section, Children Hospital, Quetta, Pakistan; 2 Centre for Advanced Studies in Vaccinology and Biotechnology (CASVAB) University of Balochistan, Brewery Road, Quetta-87300, Balochistan, Pakistan; 3 Department of Microbiology, University of Balochistan, Quetta, Pakistan; 4 Agriculture University Peshawar, Khyber Pakhtunkhwa, Pakistan

**Keywords:** Pediatric, Malaria, Falciparum, Prevalence, Vivax, Pakistan

## Abstract

**Background:**

Malaria is one of the most devastating protozoal diseases in under developing countries like Pakistan where health facilities are scarce. It is the second most frequently reported disease with 4.5 million suspected cases in Pakistan. The current study was designed to determine the incidence of pediatric malaria in Quetta, Balochistan.

**Methods:**

The study was conducted at Children Hospital Quetta (CHQ) during July 2011march 2012. Blood samples were collected from 3418 clinically suspected and were evaluated using thin and thick blood films stained with Giemsa stain.

**Results:**

Out of 3418 total of 230 (6.72%) children were found positive for any of the malarial parasitic infestation. *Plasmodium vivax* was observed to be more common 54.34% (n= 125/230) than *P. falciparum* 44.78% (n = 103/230). Male children were 65.21% (150/230) i.e. two times more commonly affected than female 34.78% (80/230) children. The prevalence among age groups was 7.41% (n = 89/1200) in preschool-aged children aged 1-5 years, 7.11% (n = 75/1054) in school-aged children aged 6—10 years while 6.78% (n = 46/678) in 11-15 years-old children, and 6.66% (n = 20/300) in >15 year-olds children. Peak prevalence was noted in summer and mild in winter. Mixed infection of (0.86%: 2/230) *P. vivax* and *P. falciparum* was also observed in two cases although no case of *P. malariae* or *P. ovale* infection was seen during entire study.

**Conclusion:**

The results reflect the higher prevalence of malaria in Quetta, Pakistan that poses a significant health threat and requires urgent attention of high-ups to launch programme to control the disease in the area.

## Introduction

Malaria is one of the most devastating diseases in the World, being endemic in more than one hundred countries. It is the second most frequently observed disease in under-developed countries. Malaria remains serious threat to public health in most of the South East Asian countries (1). Malaria is caused by number of *Plasmodium* species but the most dangerous and common species are *P. falciparum* and *P.vivax*. (2). Although all geographical regions are equally involved but populations living in sub-Saharan Africa have the highest risk of acquiring malaria. The prevalence in African children is much higher as 81.9% children were affected in Nigeria (3).

Children of all ages are prone to malaria but under age of 5 years are most affected and 86% of deaths caused by malaria reported were under age of 5years.With increasing awareness and effective control measures it is estimated that incidence of malaria has reduced globally by 17% since 2000.World Health Organization estimated that 216 million clinical malarial cases occur worldwide, with 0.655million deaths mostly among African children (4).

Pakistan is among the countries where malaria continues to be a major public health problem having extensive agricultural practices. Vast irrigation network and monsoon rains contribute to enhance the malarial potential in many areas (5). In 2004, lower malaria incidence was confined to Punjab and Azad Kashmir (AJK) whereas Balochistan and Federally Administered Tribal Areas (FATA) were reported with highest malarial incidence, while Sindh and Khyber Pakhtunkhwa were reported with moderate incidence (06). At least 39 districts, mainly from Balochistan and Sindh have been classified as high risk areas (7). In Quetta 34.8% positive smears were found, with 66.8% *P. vivax* and 30.7% *P. falciparum* (8). Pakistan is tropical agricultural country with more than 65% of the populations in rural settings, where improper waste disposal and scarcity of health facilities favors the malaria. The children have great share in the population and having generally careless life style.

This study attempts to assess the prevalence of pediatric malaria in Quetta, Pakistan.

## Materials and Methods

### Study Area

Quetta is the most populated city and provincial capital of the Balochistan Province of Pakistan with a large border at south with Islamic republic of Iran and to north with Afghanistan. The city is situated at an average elevation of 1,680 meters above sea level, making it Pakistan's only high-altitude major city. The population in 2008 was estimated to be 1.14 million. Quetta features a continental and semi-arid climate with significant variations between summer and winter temperatures. Unlike most of Pakistan, however, Quetta does not have monsoon but it experiences few spells of rainfall in winter season. Most of the population is scattered and the rural settings especially are still lacking basic living facilities.

### Study population

The study was conducted in July 2011-March 2012 at the Children Hospital Quetta (CHQ). Children less than 15 years of age and over >15 years-olds (up to 18 Years, adolescents), from different localities of Quetta were involved. Children clinically suspected for malaria with chill, shivering and febrile episodes were recruited in this study and were divided into five groups viz, <1, 1-5, 6-10, 11-15 and >15 years respectively. Most of the students with >5 years age were school going belongings to rural settings of the city, although a small number of children also were from other parts of the province.

### Collection and processing of the samples

For all clinical suspected patients following verbal consent, blood was drawn with aseptically and was transferred into sterile anticoagulant test tubes. Both thin and thick blood films were prepared and stained with Giemsa stain. The slides were air dried and observed under microscope for any plasmodial specie.

### Statistical Analysis

The data collected were subjected to statistical analysis using chi square test in SPSS 16 windows****.

## Results

Total of 3418 patients with febrile episodes and clinically suspected for malaria were included in this study period. Of them 1914 patients were male while 1504 were female children. All the patients were categorically classified into different age groups. Two hundred and thirty patients were found positive for malarial parasites. *P. vivax* was observed more commonly as 125 samples were positive, while *P. falciparum* was observed in 103 cases.

The prevalence rate of *Plasmodium* slide positivity among the age groups (Table 1) was 7.41% (89/1200) in preschool-aged children aged >1—5 years, 7.11% (75/1054) in school-aged children aged 5—10 years, 6.78% (46/678) in 10-15 years-old children, and 6.66% (20/300) in over >15 year-olds.

**Table 1:** Age wise prevalence of pediatric malaria in Quetta, Pakistan

Age (yr)	No. of subjects	Positive Subjects n (%)	*P. vivax* n (%)	*Positive cases P. falciparum* n (%)	*Mixed Infection* n (%)
**0—1**	186	---	---	---	---
**1—5**	1200	89 (7.41)	45 (50.56)	44 (49.43)	-----
**6—10**	1054	75 (7.11)	40 (53.33)	34 (45.33)	01 (1.33)
**11—15**	678	46 (6.78)	30 (65.21)	16 (34.78)	
>**15 up to 18**	300	20 (6.66)	10 (50.00)	09 (45.00)	01 (5.00)
**Total**	3418	230	125 (54.34)	103 (44.78)	02 (0.86)

No case was confirmed in infants (0–1) years. Male children were (65.21%:150/230) two times more commonly affected than female (34.78%: 80/230) children (Table 2).

**Table 2:** Sex wise prevalence of pediatric malaria in Quetta, Pakistan

Sex	No. of Patients	Positive	*P. vivax* n (%)	*Positive cases P. falciparum* n (%)	Mix Infection n (%)
**Male**	1914	150	77 (51.33)	71 (47.33)	02 (1.33)
**Female**	1504	80	48 (60.00)	32 (40.00)	---
**Total**	3418	230	125 (54.34)	103 (44.78)	02 (0.86)

The disease was predominantly more in summer than winter season as more number of cases was recorded during hot months. No case of *P. malariae* or *P. ovale* infection was seen during present study (Table 3).

**Table 3:** Month wise prevalence of pediatric Malaria in Quetta, Pakistan

Months	Total subjects	Total Positive	*P. vivax*	*P. falciparum*
**July**	320	17	7	10
**August**	45	38	18	20
**September**	512	78	38	40
**October**	454	39	19	20
**November**	416	22	10	12
**December**	322	11	4	07
**January**	348	11	5	06
**February**	250	03	00	03
**March**	340	11	04	07
**Total**	3418	230	105	125

There was no significant difference in the prevalence of malaria in different age groups. Similarly, the prevalence of *P. falciparum* was also insignificant in different age group children.

## Discussion

In the present study, the overall prevalence of 6.72% pediatric malaria was recorded which is close in agreement with other reports collected from Bunir (KPK, Pakistan) and Muzaffar Garh, Pakistan (9, 10). Similarly, a close prevalence of malaria was also reported from various districts of the Balochistan, Pakistan viz, 5.7%, 4.7% and 5.7% in Lasbella, 5.3%, 6.6% and 17.5% in Mastung and 7.3%, 7.5% and 6.8% in Sibi in three consecutive years (11). However, a much higher prevalence of 34.8% was reported from Quetta during1994-1998 as reported by (8). Similarly less prevalent of 16.2% and 15.4% was reported from the rural and urban areas of Quetta district, in 2003 and 2004, respectively (12, 13). Currently even lower pediatric typhoid prevalence of 3.06% has been reported from Bannu District of Pakistan (14). This might be due to awareness among children and relatively best sanitary measures in the area during current years. The present study reflects higher prevalence of *P. vivax* (54.34%) as compared to *P. falciparum* (44.78%). Similar findings were recorded in different districts of Balochistan and Abbotabad respectively (15, 16).

The prevalence of *Plasmodium* was higher in the two age groups (pre-school children aged 1-5 years and school-aged children aged (6-10 years) than in the other two age groups (children aged >10—15 years and adults over >15 years). No case was identified in the (infants, 0-1) first year of life. Similar pattern was seen in India, where peak incidence was in 5-9 year-old children (17).

Muhammad & Hussain (9) also reported higher rate of infection (11.58%) in the age group 1-10 year-olds from District Buner, Khyber Pakhtunkhwa, Pakistan. Similarly higher prevalence in school age group (5-10 yr) in Larkana, Pakistan was recorded (18). This may be attributed to greater mobility and outdoor activities of children in this age group, hence more chances of exposure to mosquitoes leading to malaria.

Regarding sex, male children were two times more commonly affected (65.21%) than female (34.78%) in our study which is supported by many scientists in different parts of the country. Moreover, Sahar et al. (19) reported from Muzaffar Garh, Punjab-Pakistan that males were significantly more likely to get the disease than the females. This male predominance may be due to more outdoor activities of males in comparison to females. Hence, they are more prone to the disease.

In our study prevalence of *Plasmodium* exhibited increasing pattern from July and August (summer), peaked in September and remained moderate till (mild weather) October-November while lower rates were noted in winter (December-March). Mahmood (20) and Farogh, et al. (21) also reported similar findings from Karachi and Bahawalpur, Pakistan. The possible reason could be the fact that these months experience the post monsoon period in most parts of the Pakistan in which the ambient climatic conditions favor mosquito breeding. However, unlike most of Pakistan, Quetta does not have a monsoon with heavy rainfall but the high population of mosquitoes is likely to occur during summer (July -September) in Quetta. No case of *P. malariae* or *P. ovale* infection was observed during present study. This statement was supported by different researchers from different areas of Pakistan. (16, 17, 21, 22).

## Conclusion

The prevalence rate of 6.72% malaria with predominance of *P. vivax* (54.34%) in this study was observed in school age (5–15) years children. The disease was two folds more in male children and relatively higher rate of positivity was recorded in descending summer months. Malaria is difficult to eradicate, however, measures can be taken to minimize the chances of spread of the disease.
